# Chrysin Inhibits TAMs-Mediated Autophagy Activation via CDK1/ULK1 Pathway and Reverses TAMs-Mediated Growth-Promoting Effects in Non-Small Cell Lung Cancer

**DOI:** 10.3390/ph17040515

**Published:** 2024-04-17

**Authors:** Xinglinzi Tang, Xiaoru Luo, Xiao Wang, Yi Zhang, Jiajia Xie, Xuan Niu, Xiaopeng Lu, Xi Deng, Zheng Xu, Fanwei Wu

**Affiliations:** 1 Central Lab, The Seventh Clinical Medicial College of Guangzhou University of Chinese Medicine, Shenzhen 518000, China; zelda47@126.com (X.T.); lxiaoru723@126.com (X.L.); 2 Department of Basic Theory of TCM, Guangzhou University of Chinese Medicine, Guangzhou 510330, China; 18487288184@163.com; 3 Department of Psychology, School of Public Health and Management, Guangzhou University of Chinese Medicine, Guangzhou 510330, China; 4 Department of Classic Traditional Chinese Medicine, The Seventh Clinical Medicial College of Guangzhou University of Chinese Medicine, Shenzhen 518000, China; jiajiaxiedr@126.com (J.X.); minniekiss.9@163.com (X.N.); luxiaopengdr@163.com (X.L.); deng.x@163.com (X.D.)

**Keywords:** non-small cell lung cancer, TAMs, autophagy, CDK1/ULK1 signaling pathway, chrysin

## Abstract

The natural flavonoid compound chrysin has promising anti-tumor effects. In this study, we aimed to investigate the mechanism by which chrysin inhibits the growth of non-small cell lung cancer (NSCLC). Through in vitro cell culture and animal models, we explored the impact of chrysin on the growth of NSCLC cells and the pro-cancer effects of tumor-associated macrophages (TAMs) and their mechanisms. We observed that M2-TAMs significantly promoted the growth and migration of NSCLC cells, while also markedly activating the autophagy level of these cells. Chrysin displayed a significant inhibitory effect on the growth of NSCLC cells, and it could also suppress the pro-cancer effects of M2-TAMs and inhibit their mediated autophagy. Furthermore, combining network pharmacology, we found that chrysin inhibited TAMs-mediated autophagy activation in NSCLC cells through the regulation of the CDK1/ULK1 signaling pathway, rather than the classical mTOR/ULK1 signaling pathway. Our study reveals a novel mechanism by which chrysin inhibits TAMs-mediated autophagy activation in NSCLC cells through the regulation of the CDK1/ULK1 pathway, thereby suppressing NSCLC growth. This discovery not only provides new therapeutic strategies for NSCLC but also opens up new avenues for further research on chrysin.

## 1. Introduction

Lung cancer is a prevalent malignancy globally and ranks among the leading causes of cancer-related deaths. In 2020, there were nearly 2.21 million new cases and approximately 1.8 million deaths globally attributed to lung cancer [1]. Non-Small Cell Lung Cancer (NSCLC) is the predominant type, accounting for 80–85% of all lung cancers, including lung adenocarcinoma and lung squamous cell carcinoma [2]. Currently, lung cancer has entered the era of precision therapy. Through the detection of multiple molecular and immune biomarkers, including EGFR, ALK, and PD-L1, among others, doctors can selectively choose targeted therapies such as small-molecule tyrosine kinase inhibitors (TKIs) and PD-1/PD-L1 inhibitors based on the molecular characteristics of individual patients [3,4]. This approach aims to improve patient survival rates and quality of life. However, existing targeted and immune therapies are generally effective only for patients testing positive for the corresponding targets, and there are issues of acquired resistance. Therefore, further in-depth molecular research on NSCLC, revealing its developmental molecular mechanisms and exploring new treatment strategies, is of significant clinical importance [5]. The tumor microenvironment (TME) is a complex ecosystem surrounding tumor cells, comprising various components such as cancer cells, immune cells, blood vessels, and stromal elements [6]. Among these components, Tumor-Associated Macrophages (TAMs) play a significant role in the TME. TAMs can be divided into two forms: M1 macrophages with anti-tumor functions and M2 macrophages with pro-tumor functions [7]. In the TME, TAMs predominantly display the M2 phenotype, promoting tumor growth and invasion by secreting factors such as vascular endothelial growth factor and matrix metalloproteinases [8]. However, the exact roles and mechanisms of TAMs in the development of lung cancer are not yet fully understood.

Autophagy is a self-protective mechanism widely present in eukaryotic cells, playing a crucial role in maintaining cellular homeostasis [9]. Autophagy has dual functions in tumorigenesis and development. It can degrade damaged organelles and proteins, thus inhibiting tumor initiation; however, it can also provide energy for tumor cells, promoting their growth and survival [10]. Earlier studies have demonstrated that TAMs can influence the autophagy of surrounding cells by secreting various cytokines and signaling molecules, such as transforming growth factor-β (TGF-β) and interleukin-10 (IL-10) [11,12]. For instance, Fu et al. found that TAMs could activate autophagy in hepatocellular carcinoma cells, leading to reduced sensitivity to the inhibitory effects of oxaliplatin [13]. Conversely, autophagy may also modulate the function and phenotype of TAMs, thereby altering the tumor microenvironment. For example, it has been discovered that tumor-released autophagosomes (TRAPs) can promote the polarization of macrophages into the M2 phenotype, thus facilitating tumor growth [14]. Therefore, targeting both autophagy and TAMs simultaneously may represent a novel anti-tumor strategy.

Chrysin is a natural flavonoid compound widely present in a range of plants, including passion fruit, honeycomb, and mushrooms [15]. Owing to its wide range of biological functions, which encompass antioxidant, anti-inflammatory, and anti-tumor effects, it has garnered significant attention in recent years [16]. As a novel natural anti-cancer drug, chrysin has demonstrated promising anti-tumor effects in both laboratory and clinical trials [17]. Research findings indicate that chrysin can inhibit the growth and metastasis of tumor cells by modulating multiple signaling pathways, including NF-κB, MAPK, PI3K/AKT, and ROS pathways [18,19,20]. Additionally, chrysin can induce apoptosis in tumor cells, enhance chemotherapy, and reverse drug resistance [21]. Currently, the anti-lung cancer activity of chrysin has been confirmed [22], yet its exact mechanism of action remains unclear, limiting its clinical applications. Therefore, in-depth research into the anti-tumor mechanisms of chrysin will help unlock its potential as a novel anti-cancer drug.

In this study, we utilized chrysin to intervene in NSCLC cells. Our experiments revealed that chrysin can inhibit the transformation of TAMs into the M2 phenotype and reverse TAMs-induced activation of autophagy in NSCLC cells, thereby suppressing the proliferation of the cancer cells in vitro and in vivo. Furthermore, our investigations, integrating network pharmacology and experimental validation, have uncovered the capability of chrysin to potentially inhibit TAMs-mediated autophagy in lung cancer cells by modulating the CDK1/ULK1 signaling pathway.

## 2. Results

### 2.1. M2-TAMs Promoted the Growth and Migration of NSCLC Cells 

**Figure 1:**
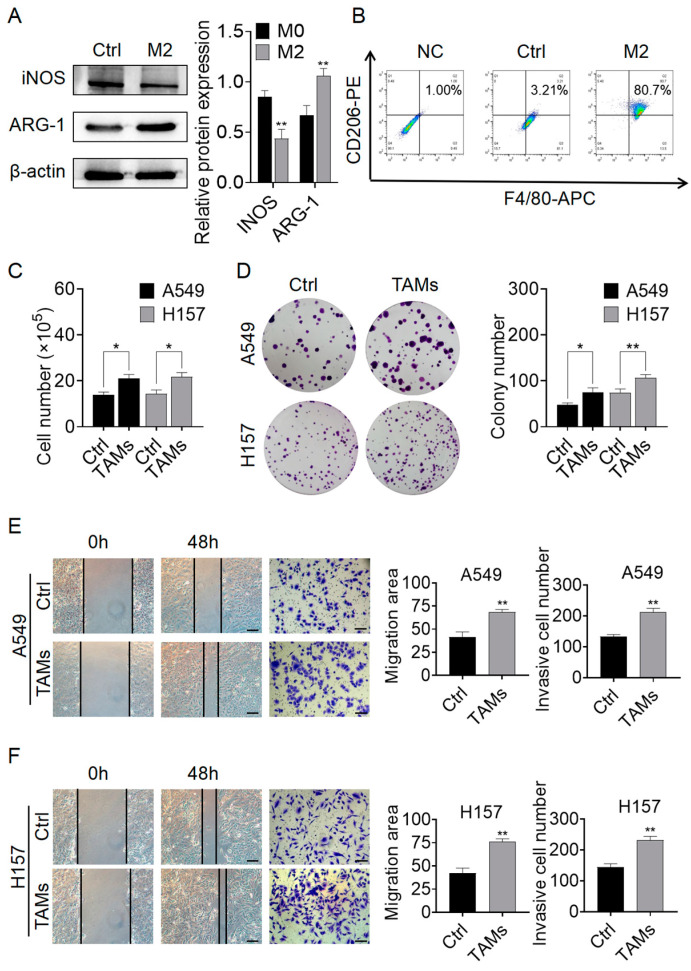
The impact of M2-TAMs on the proliferation and migration abilities of NSCLC cells was assessed. (**A**) Macrophage polarization was detected using WB; (**B**) macrophage polarization was evaluated through flow cytometry; (**C**) cell counting was used to examine the influence of M2-TAMs on the proliferation ability of A549 and H157 NSCLC cells; (**D**) clone formation experiment was conducted to assess the effect of M2-TAMs on the clonogenic potential of A549 and H157 NSCLC cells; (**E**) scratch and transwell assays were performed to investigate the influence of M2-TAMs on the migratory ability of A549 cells; (**F**) scratch and transwell assays were carried out to determine the impact of M2-TAMs on the migration ability of H157 cells. Scale bars in the images for both scratch and transwell assays represent 100 μm. All values are presented as mean ± SD (*n* = 3, * *p* < 0.05, ** *p* < 0.01 vs. Ctrl group).

First, we established the M2-TAMs model through cytokine induction and verified it using Western blot (WB) and flow cytometry. As shown in Figure 1A,B, the M2-TAMs exhibited reduced levels of iNOS protein and elevated levels of ARG-1 protein. iNOS is a marker of M1-TAMs, while ARG-1 is a marker of M2-TAMs [23]. Around 80% of cells were found to be positive for both F4/80 and CD206 markers, indicating a successful construction of the M2-TAMs (referred to as TAMs in the following text) model. Next, we used the supernatant of TAMs for A549 and H157 cell lines to investigate the effect of M2-TAMs on NSCLC cell growth. The cell counting results showed that the proliferation of both NSCLC cell lines significantly increased under the influence of TAMs compared to the Ctrl group (Figure 1C). Clonogenic assay results also suggested that TAMs promoted the clonal formation of both NSCLC cell lines, with a significant increase in both clone size and number in the TAMs group (Figure 1D). Subsequently, migration and invasive detection revealed that the healing area and the number of migrating cells of NSCLC cells were significantly increased in the TAMs group compared to the Ctrl group (Figure 1E,F). These results indicate that TAMs significantly enhance the growth and migration capabilities of NSCLC cells. 

### 2.2. Chrysin Reversed the Pro-Tumor Effects of TAMs 

**Figure 2:**
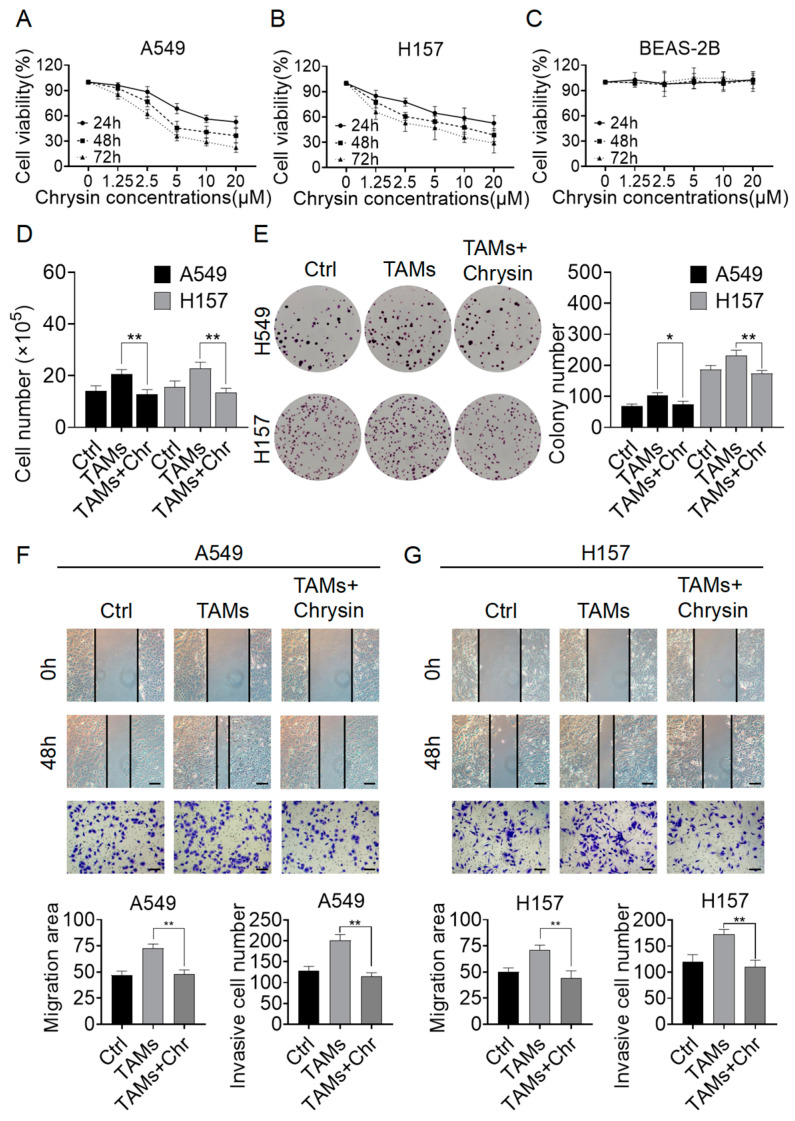
Chrysin inhibited the growth of NSCLC cells and reversed the pro-tumor effect of TAMs. (**A**,**B**) CCK-8 assay demonstrated that chrysin (0–20 µM) suppressed the proliferation of A549 and H157 cells; (**C**) CCK-8 assay indicated that chrysin did not exhibit a significant inhibitory effect on the normal human lung epithelial cell BEAS-2B; (**D**) cell counting and (**E**) colony formation assay showed that chrysin (8 µM) attenuated the TAMs-induced proliferation of both NSCLC cell lines; (**F**) scratch and transwell assays revealed that chrysin (8 µM) reversed the TAMs-induced migration-promoting effect on A549 cells; (**G**) scratch and transwell assays demonstrated that chrysin (8 µM) also reversed the TAMs-induced migration-promoting effect on H157 cells. Scale bars in the images for both scratch and transwell assays represent 100 μm. All values are presented as mean ± SD (*n* = 3, * *p* < 0.05, ** *p* < 0.01 vs. TAMs group).

Subsequently, we treated A549 and H157 cells, as well as normal human lung epithelial cells BEAS-2B, with chrysin to assess its effects on cell growth. The CCK-8 results demonstrated that chrysin inhibited the proliferation of both NSCLC cell lines in a dose- and time-dependent manner (Figure 2A,B). Furthermore, chrysin did not show significant toxicity towards normal human lung epithelial cells (Figure 2C). The IC50 values of chrysin in the A549 cell line were 17.59 μM, 7.29 μM, and 4.11 μM at 24 h, 48 h, and 72 h, respectively. In the H157 cell line, the IC50 values were 20.65 μM, 7.83 μM, and 3.70 μM, respectively. Next, we treated A549 and H157 cells with supernatant from TAMs in combination with chrysin at a concentration of 8 μM for cell counting and clonogenic assays. The results showed that upon the addition of Chrysin, both the cell number and the size and quantity of cell clones decreased significantly compared to the TAMs group (Figure 2D,E). Following that, migration and invasive detection were performed, demonstrating a decrease in the healing area and the number of migrating cells of NSCLC cells upon treatment with chrysin (Figure 2F,G). These results indicate that chrysin not only inhibits the growth of NSCLC cells but also reverses the promoting effect of TAMs on the growth and migration capabilities of NSCLC cells.

### 2.3. Chrysin Reversed the Pro-Tumor Effects of TAMs by Inhibiting Autophagy Mediated by TAMs 

#### 2.3.1. Chrysin Inhibited Macrophage Polarization towards the M2 Phenotype and Suppressed Autophagy Activation Mediated by TAMs

**Figure 3:**
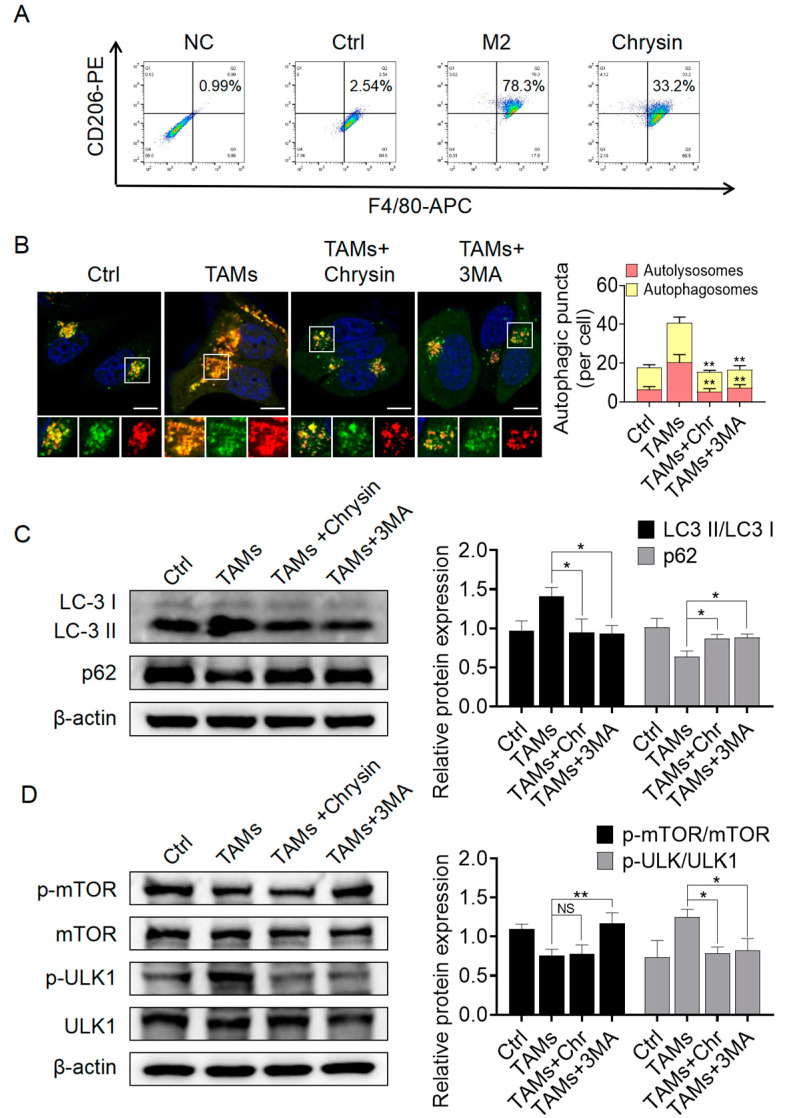
Chrysin inhibited the polarization of macrophages towards the M2 phenotype and inhibited autophagy activation mediated by TAMs. (**A**) Flow cytometry results demonstrated that chrysin inhibited macrophage polarization into M2-TAMs; (**B**) LC3-mRFP-GFP lentiviral vectors transfection was used to investigate the effect of chrysin (8 µM) and 3-MA (10 mM) on autophagic flux in A549 cells treated with TAMs-conditioned media; (**C**) Western blotting assessed the expression of autophagy-related proteins, LC3 and p62, in A549 cells treated with TAMs-conditioned media and treated with chrysin (8 µM) or 3-MA (10 mM); (**D**) Western blotting examined the expression of early autophagy proteins, mTOR, p-mTOR, ULK1, and p-ULK1, in A549 cells treated with TAM-conditioned media and treated with chrysin (8 µM) or 3-MA (10 mM). Scale bars in the immunofluorescence images represent 10 μm. All values are presented as mean ± SD (*n* = 3, * *p* < 0.05, ** *p* < 0.01 vs. TAMs group, NS indicates nonsignificant).

Then, we treated THP-1 cells with chrysin to assess its effects on macrophage differentiation. Flow cytometry results showed that under chrysin treatment, the percentage of double-positive cells for F4/80 and CD163 decreased from 78% in the control group to 33%, indicating that chrysin can inhibit the polarization of macrophages into the M2 phenotype (Figure 3A). In addition, we established an A549 and M2-TAMs co-culture system to investigate the impact of chrysin on TAM polarization in the presence of both A549 cells and M2-TAMs. The results (Supplementary Figure S1) indicated that the addition of chrysin inhibited the polarization of M2-TAMs, resulting in a decrease of about 20% in the proportion of double-positive cells compared to the M2+A549 group. This further confirms the inhibitory effect of chrysin on the polarization of TAMs towards the M2 phenotype. To investigate the effects of TAMs and chrysin on autophagy in NSCLC cells, we first transfected A549 cells with LC3-mRFP-GFP lentiviral vectors to visualize the fusion of autophagosomes with lysosomes using fluorescent protein signals. When autophagy is activated, a significant fusion between autophagosomes and lysosomes occurs, leading to the quenching of the green fluorescent protein (GFP-LC3) signal within autophagosomes in the acidic lysosomal environment, resulting in the display of only the red fluorescent protein (mRFP-LC3), while the yellow puncta decrease [24]. The results, as shown in Figure 3B, revealed that TAMs treatment increased the number of red and yellow puncta in A549 cells compared to the control group, indicating the activation of autophagy by TAMs. Upon addition of chrysin, the number of red and yellow puncta decreased significantly, consistent with the results obtained by adding an early autophagy inhibitor, 3-MA. These findings suggest that TAMs can activate autophagy in NSCLC cells, while chrysin can suppress TAMs-mediated autophagy activation and inhibit early autophagy. We further conducted Western blot experiments to assess the expression levels of autophagy-related proteins LC3 and p62 in NSCLC cells under these conditions. The transition from LC3-I to LC3-II is recognized as an indicator of autophagy, and p62, serving as a substrate for autophagy, undergoes degradation throughout the autophagic process [25]. Therefore, LC3-II expression decreases, and p62 expression increases when autophagy is inhibited [26]. As shown in Figure 3C, the administration of TAMs resulted in a notable elevation of the LC3-II/LC3-I ratio and a reduction in p62 levels in A549 cells in comparison to the Ctrl group. Furthermore, upon addition of chrysin, the LC3-II/LC3-I ratio decreased, while p62 levels increased compared to the TAMs group, consistent with the effects of the early inhibitor, 3-MA. These Western blot results further confirmed that chrysin could reverse TAMs-mediated autophagy activation. Next, we examined the expression status of two early autophagy markers, mTOR and ULK1, and their phosphorylated proteins in A549 cells treated with chrysin. The Western blot results (Figure 3C) showed that TAMs inhibited the phosphorylation of mTOR in A549 cells and significantly promoted the phosphorylation of ULK1. Upon addition of chrysin and 3-MA, the phosphorylation level of ULK1 decreased significantly. However, interestingly, unlike 3-MA, chrysin did not reverse the decrease in mTOR phosphorylation caused by TAMs, and the phosphorylation level of mTOR showed no significant change compared to the TAMs group. Thus, we speculate that chrysin directly inhibits TAMs-mediated NSCLC cell autophagy activation by suppressing the phosphorylation of ULK1 without affecting mTOR expression. 

#### 2.3.2. Chrysin Reversed the Pro-Cancer Effects of TAMs by Inhibiting Autophagy 

**Figure 4:**
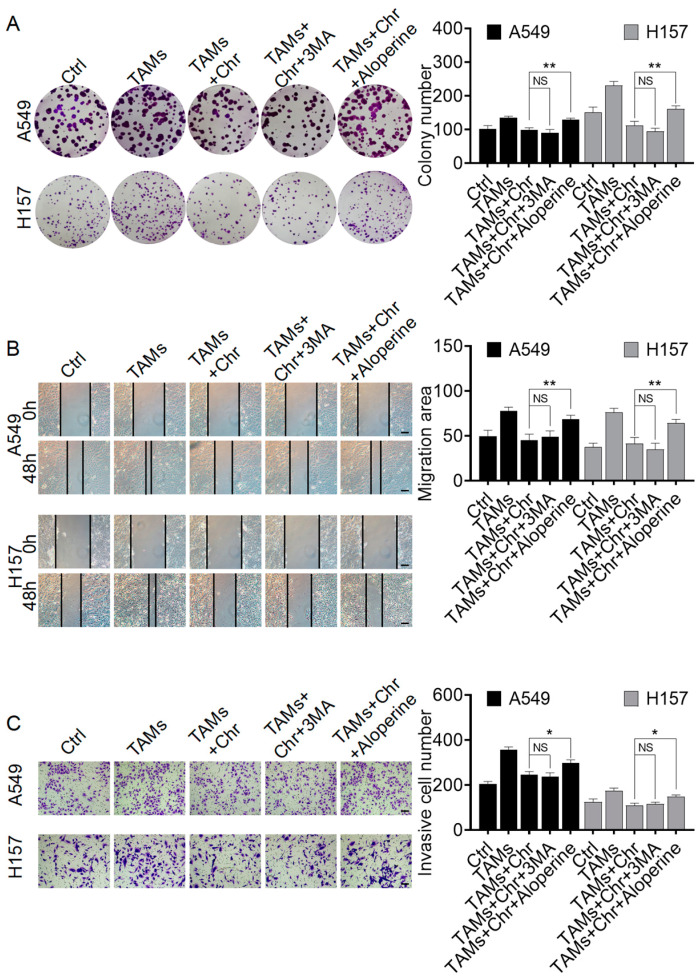
Chrysin reversed the pro-cancer effects of TAMs by inhibiting autophagy. (**A**) The colony formation assay reflected the effects of chrysin (8 µM), 3-MA (10 mM), and aloperine (100 µM) on the clonogenicity of A549 and H157 cells; (**B**) representative images of scratch assays conducted on A549 and H157 cells after the indicated treatments; (**C**) representative images of scratch and transwell assays conducted on A549 and H157 cells after the respective treatments. Scale bars in the images for both scratch and transwell assays represent 100 μm. All values are presented as mean ± SD (*n* = 3, * *p* < 0.05, ** *p* < 0.01 vs. TAMs+ chrysin group, NS indicates nonsignificant).

To further investigate the critical mechanism by which chrysin reverses the pro-cancer effects of TAMs, we used the autophagy inhibitor 3-MA and the autophagy activator aloperine to treat A549 and H157 cells and performed colony formation, scratch, and transwell experiments for validation. As shown in Figure 4A, the addition of aloperine significantly increased the number and size of cell colonies compared to the TAMs+ chrysin group. However, the addition of 3-MA did not affect the inhibitory effect of chrysin on cell cloning ability. Similar results were observed in the scratch and transwell experiments (Figure 4B,C). These findings indicate that the pro-cancer effects of TAMs are similar to 3-MA and opposite to aloperine. Therefore, chrysin reverses the pro-cancer effects of TAMs by inhibiting autophagy. 

### 2.4. The Target Exploration of Chrysin 

**Figure 5:**
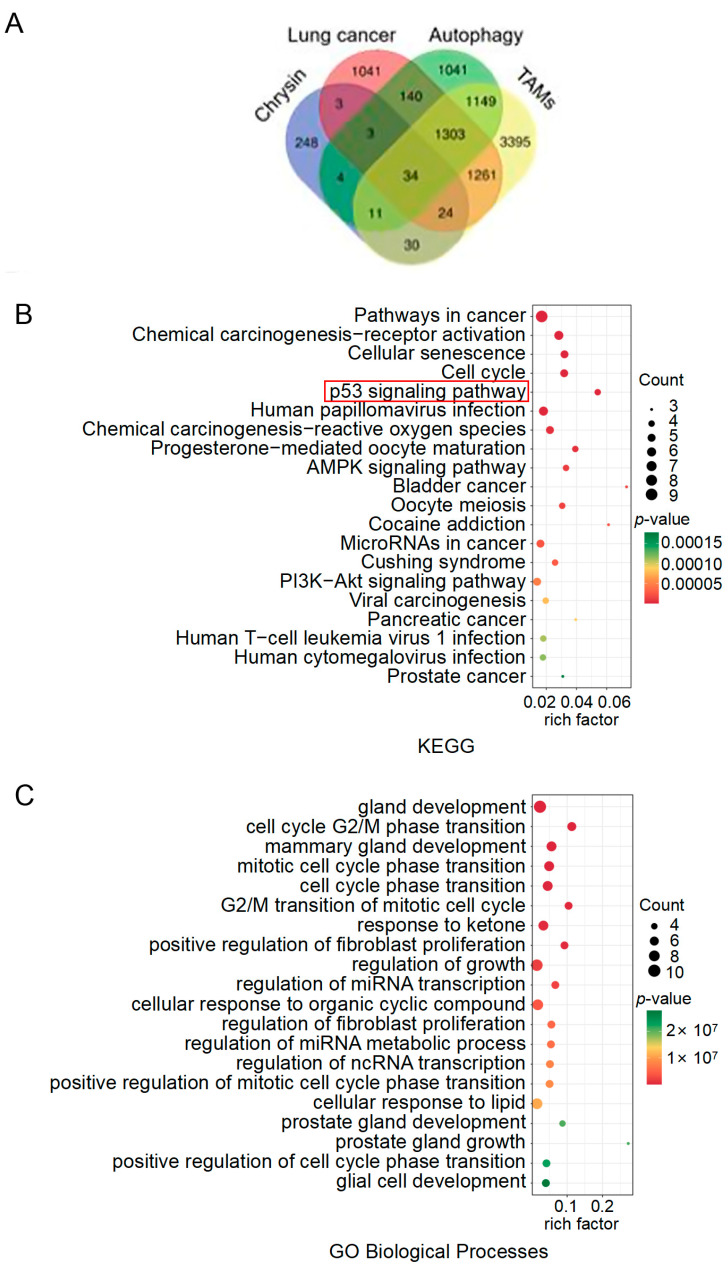
KEGG pathway enrichment and GO analysis for “Chrysin”, “Non-small Cell Lung Cancer”, “Tumor Associated Macrophages”, and “Autophagy”. (**A**) Venn diagram illustrating the overlap among the targets of “Chrysin”, “Non-small Cell Lung Cancer”, “Tumor Associated Macrophages”, and “Autophagy”; (**B**) bubble chart showing the KEGG terms for enrichment analysis; (**C**) bubble chart depicting the GO biological processes analysis.

**Figure 6:**
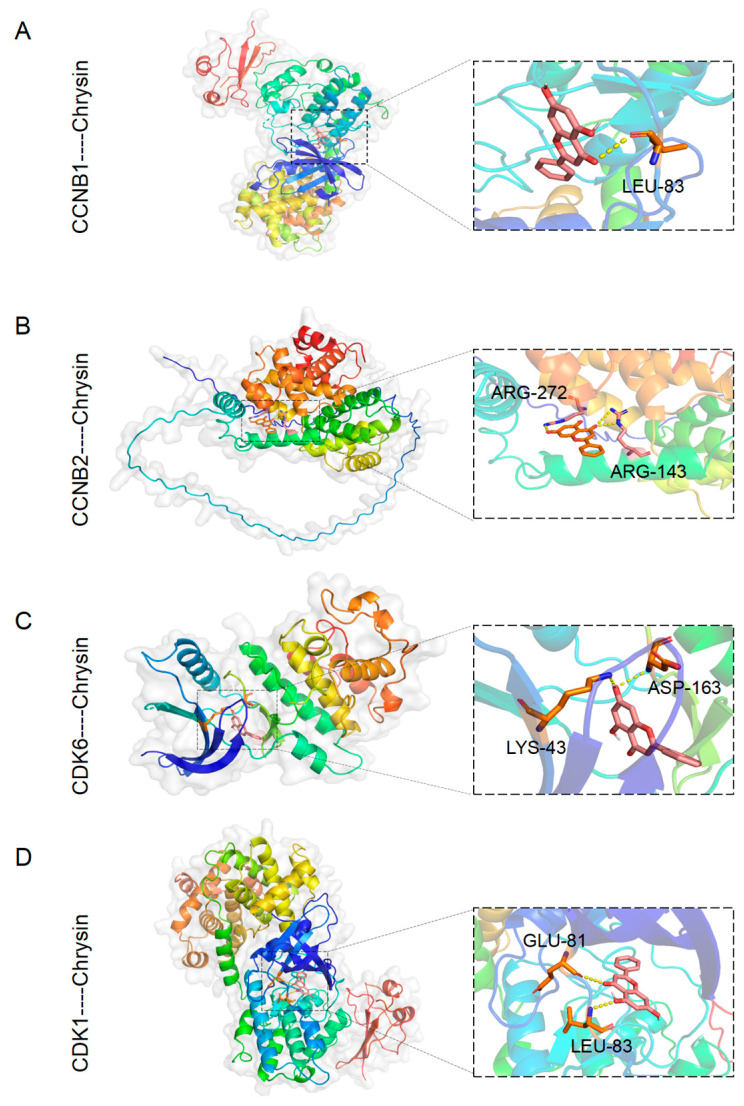
Molecular docking. (**A**) The image shows chrysin docked into the active site of CCNB1; (**B**) the image depicts chrysin docked in the active site of CCNB2; (**C**) the image displays chrysin docked within the active site of CDK6; (**D**) the image illustrates chrysin docked at the active site of CDK1.

To identify potential targets responsible for the actions of chrysin mentioned above, we first conducted bioinformatics analysis and obtained 7207 protein targets of “Tumor Associated Macrophages”, 3809 targets of “Non-small Cell Lung Cancer”, 3685 targets of “Autophagy”, and 358 targets of “Chrysin” from network databases. There were 34 common target genes among these four categories (Figure 5A). KEGG pathway analysis revealed 37 relevant pathways, with the top 5 pathways with the smallest *p*-values being Pathways in cancer, chemical carcinogenesis—receptor activation, cellular senescence, cell cycle, and p53 signaling pathway (Figure 5B). Apart from the p53 signaling pathway, the other four pathways had an overly broad scope; thus, the p53 signaling pathway was selected for further investigation. GO Biological Processes mainly included gland development, cell cycle G2/M phase transition, mammary gland development, mitotic cell cycle phase transition, and cell cycle phase transition (Figure 5C). Subsequently, we conducted molecular docking studies to explore the potential interactions between chrysin and the p53 signaling pathway (Figure 6). According to the results of the KEGG pathway enrichment analysis, chrysin primarily influenced four targets in the p53 signaling pathway: CCNB1, CCNB2, CDK1, and CKD6 (Supplementary Table S1). The molecular docking results indicated that chrysin could successfully dock with all four targets, with the highest docking score observed for CDK1 at 9.6 points (Supplementary Table S2). 

### 2.5. Chrysin Inhibits Autophagy through the CDK1/ULK1 Pathway

**Figure 7:**
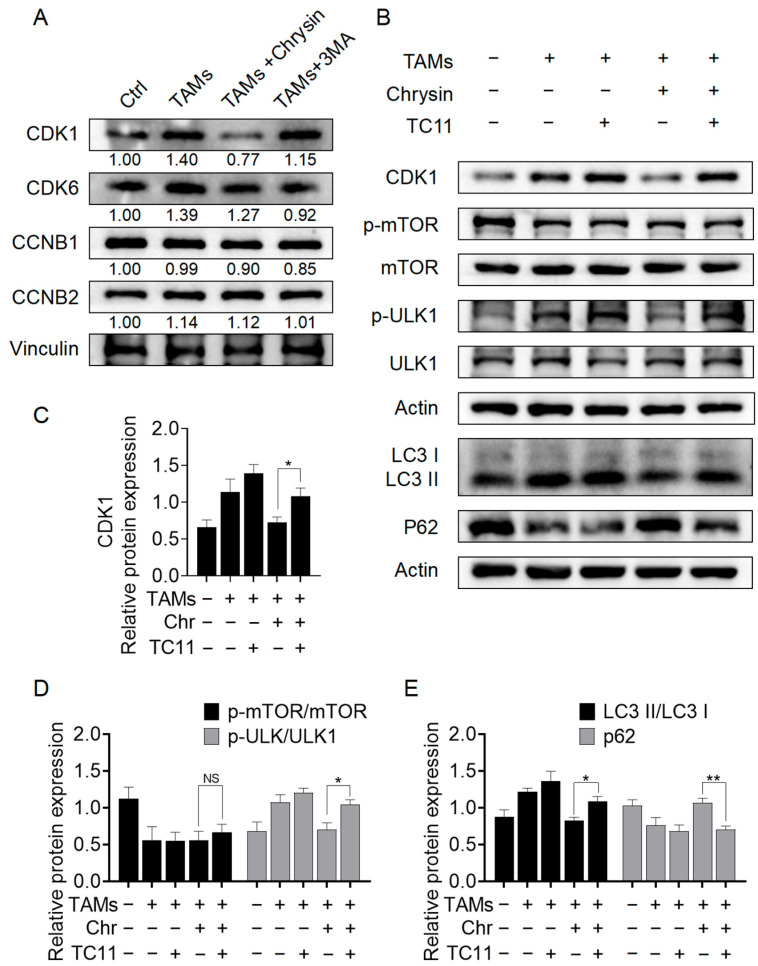
Chrysin inhibited autophagy through the CDK1/ULK1 pathway. (**A**) The expression of CDK1, CDK6, CCNB1, and CCNB2 in A549 cells was detected via Western blot after the specified treatments. (**B**) The expression of CDK1, LC3, p62, mTOR, p-mTOR, ULK1, and p-ULK1 in A549 cells was examined by Western blot after treatment with TAMs and exposure to chrysin (8 μM) and/or TC11 (5 μM). (**C**) Histogram of the grayscale values for CDK1 in (**B**). (**D**) Histogram of the grayscale values for p-mTOR/mTOR and p-ULK/ULK1 in (**B**). (**E**) Histogram of the grayscale values for LC3 II/LC3 I and p62 in (**B**). All values are presented as mean ± SD (*n* = 3, * *p* < 0.05, ** *p* < 0.01 vs. TAMs + chrysin group, NS indicates nonsignificant).

To further validate the results obtained from prior bioinformatics analysis and molecular docking, we performed Western blotting to verify the impact of chrysin on the expression of CDK1, CDK6, CCNB1, and CCNB2 proteins in NSCLC cells. As shown in Figure 7A, upon the addition of chrysin, the elevated CDK1 levels in TAMs-induced A549 cells were significantly reversed, decreasing from 1.4-fold to 0.7-fold (fold change was calculated by comparison with the Ctrl group). The effects on CDK6, CCNB1, and CCNB2 were less pronounced compared to CDK1. Therefore, we hypothesized that the reversal of TAMs’ pro-cancer effects by chrysin may be linked to the suppression of CDK1 expression. To further confirm this, we treated A549 cells with the CDK1 activator TC11 and performed Western blotting. The results (Figure 7B–E) showed that after TC11 intervention, the decrease in CDK1 levels induced by chrysin in the cells was reversed. Additionally, upon TC11 treatment, there was a significant increase in ULK1 phosphorylation and the LC3 II/I ratio, accompanied by a noticeable decrease in p62 levels compared to the TAMs + chrysin group. These results suggest that chrysin exerts its autophagy-inhibiting effects by suppressing CDK1 and inhibiting ULK1 phosphorylation. Furthermore, CDK1 potentially interacts with ULK1 in a downstream relationship, suggesting that the CDK1/ULK1 pathway may be a crucial mechanism for chrysin to inhibit autophagy in NSCLC cells mediated by TAMs.

### 2.6. Chrysin Reversed the Pro-Cancer Effects of TAMs In Vivo 

**Figure 8:**
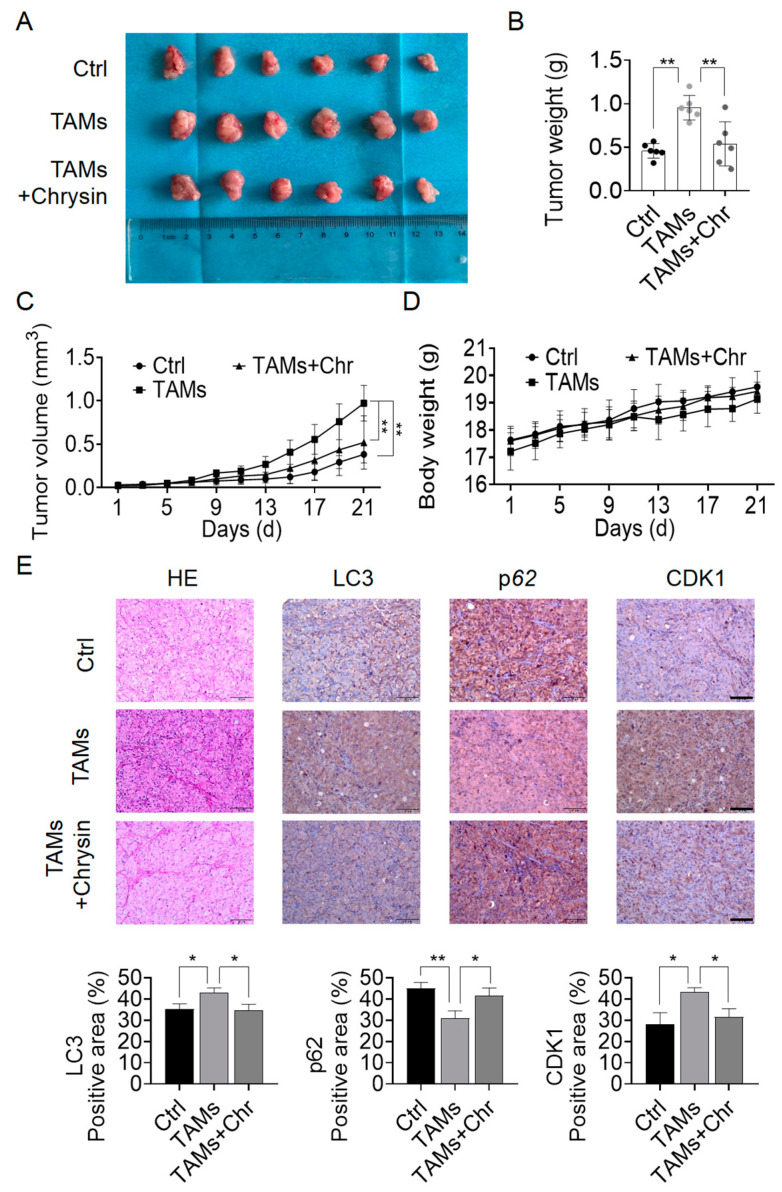
Chrysin reversed the pro-tumor effect of TAMs in vivo. (**A**) Photographic images of mouse tumors; (**B**) tumor weight of mice after treatment with TAMs and chrysin (30 mg/kg); (**C**) tumor volume change in mice over 21 days; (**D**) body weight change in mice over 21 days; (**E**) H&E staining and IHC analysis investigating the effects of TAMs and chrysin on the proliferation of lung cancer cells, as well as the expression of LC3, P62, and CDK1 proteins. Scale bars in the images for both H&E staining and IHC assays represent 100 μm. All values are presented as mean ± SD (* *p* < 0.05, ** *p* < 0.01 vs. TAMs group).

To support the findings from the in vitro studies, animal experiments were conducted for in vivo validation. We established a xenograft tumor model and administered chrysin orally to randomly grouped mice. After 21 days, the mice were harvested. Visual examination clearly demonstrated a substantial rise in tumor volume and weight in mice injected with TAMs compared to the Ctrl group. With chrysin intervention, the tumor volume and weight in the TAMs + chrysin group were markedly reduced compared to the TAMs group (Figure 8A–C). Moreover, chrysin treatment did not affect the growth of mouse body weight, indicating no apparent toxicity of chrysin to mice (Figure 8D). Results from H&E staining also indicated that chrysin could inhibit the proliferative effects of TAMs on NSCLC cells (Figure 8E). Immunohistochemistry results suggested that chrysin suppressed the expression of CDK1 and LC3, while promoting the expression of p62, consistent with the in vitro experiments (Figure 8E).

## 3. Discussion

M2-TAMs play a crucial role in tumor growth and progression as essential components of the tumor microenvironment. Their impact on tumor growth is multifaceted, with one key aspect being the promotion of tumor growth by enhancing tumor cell proliferation and invasion. TAMs can secrete various cytokines and chemokines, such as epidermal growth factor (EGF), transforming growth factor (TGF), and IL-10 (interleukin-10), which can directly stimulate tumor cell proliferation and facilitate tumor cell migration and invasion, thereby driving tumor growth and dissemination [27,28]. Furthermore, TAMs are among the principal innate immune cell populations in tumors, with M2-TAMs capable of suppressing immune surveillance, aiding tumor cell immune evasion, protecting tumors from cytotoxic immune reactions, and promoting tumor progression [29]. Additionally, TAMs influence tumor growth by modulating the autophagic process of tumor cells. Firstly, TAMs can regulate the expression of certain autophagy-related signaling molecules such as mTOR, AKT, and MAPK, thereby controlling the level of autophagy in tumor cells [30,31,32]. For instance, Vekariya et al. found that the POTE (Prostate, Ovary, Testes, and Embryo) gene in TAMs can directly influence mTOR activity, activating biological functions in tumor cell invasion [30]. Secondly, TAMs can indirectly affect tumor cell autophagy by altering the tumor microenvironment. For example, TAMs can induce tumor cell autophagy by producing inflammatory factors such as IL-6 (interleukin-6) and IL-8 (interleukin-8) [33]. These inflammatory factors have the capacity to trigger the activation of the NF-κB (nuclear factor kappa B) signaling pathway, promoting the expression of ATG1 and thereby enhancing tumor cell autophagy [34,35]. Therefore, regulating autophagy mediated by TAMs may be one of the mechanisms to prevent tumors from evading immune destruction through TAMs. In our study, TAMs significantly activated the autophagic level of NSCLC cells, promoting tumor cell growth and invasion ability. After treatment with Chrysin, the pro-cancer effects of TAMs were markedly suppressed, and the cells transitioned from high autophagic levels to low autophagic levels. This indicates that autophagy indeed serves as a critical mechanism through which TAMs promote NSCLC cell growth.

CDK1 is a pivotal regulatory protein of the cell cycle, having regulated nuclear envelope breakdown (NEB) during the early phases of mitosis [36]. A decrease in CDK1 activity facilitated premature reassembly of the nuclear envelope and exit from mitosis [37]. During the initial phases of mitosis, NEB exposes the contents of the cell nucleus to the cytoplasm, consequently influencing the occurrence of autophagy [38]. Some have posited that autophagy would be suppressed during this process to ensure the integrity of the nucleus contents [39]. However, active autophagy has also been observed during cell mitosis by others [40]. Regardless, CDK1 may still represent a crucial target for the regulation of autophagy. Odle and others revealed that during mitosis, the activity of mTORC1 was inhibited, allowing CDK1 to emerge as the main regulator of autophagy in this phase, impacting the mitotic phosphorylation of autophagic regulatory factors, including ULK1, ATG13, and ATG14. In our study, the introduction of chrysin did not alter the expression of mTOR in tumor cells; conversely, it inhibited the phosphorylation of ULK1, effectively reversing the autophagic activation state in the cells. Therefore, it may be inferred that chrysin inhibits TAMs-mediated autophagy via the CDK1/ULK1 pathway instead of the classical mTOR/ULK1 autophagy pathway. The disclosure of this mechanism also opens new avenues for the development of chrysin in cancer treatment research.

Chrysin, as a natural product, offers effective, safe, and cost-effective advantages in tumor treatment. Apart from its pharmacological effects such as anti-inflammatory [41], antioxidant [42], and lipid-lowering [43] properties, the anti-tumor activity of chrysin has also garnered attention. Lirdprapamongkol et al. found through in vivo and in vitro experiments that chrysin can inhibit breast cancer proliferation under hypoxic conditions and reduce breast cancer lung metastasis [44]. Also, chrysin could promote apoptosis in lung cancer cells and increase their sensitivity to chemotherapy drugs [45,46]. In this study, we explored the anti-tumor effect of chrysin at the molecular level through in vivo and in vitro experiments. The results indicated that chrysin can inhibit M2-TAMs-mediated autophagy through CDK1/ULK1 pathway, with no significant toxicity to normal lung epithelial cells at effective concentrations. These findings suggest that chrysin holds great potential in the precision treatment of NSCLC. Moreover, a clinical trial has introduced the application of chrysin in the treatment of metastatic colorectal cancer patients, showing that chrysin can reduce the diarrhea caused by the chemotherapy drug irinotecan [47]. Therefore, further efforts are warranted in chrysin research. If our findings regarding the impact of chrysin on the NSCLC immune microenvironment and autophagy-related molecular pathways can be confirmed through clinical trials, it could bring new detection and treatment strategies for NSCLC patients, offering new hope for extending survival and improving quality of life in NSCLC patients.

## 4. Materials and Methods

### 4.1. Cell Culture

The human non-small cell lung cell line A549 and lung adenocarcinoma cell line H157, normal human lung epithelial cell line BEAS-2B, and human monocytic cell line THP-1 were all purchased from the American Type Culture Collection (ATCC, Manassas, VA, USA). All cells were cultured in complete medium containing 10% fetal bovine serum (Gibco, Grand Island, NY, USA) and 1% penicillin-streptomycin (Gibco, Grand Island, NY, USA) and maintained in a humidified incubator at 37 °C with 5% CO_2_. A549 and BEAS-2B cells were cultured in DMEM (Gibco, Grand Island, NY, USA) medium, while H157 and THP-1 cell lines were cultured in RPMI-1640 (Gibco, Grand Island, NY, USA) medium.

### 4.2. Macrophage Induction 

THP-1 cells in suspension were seeded at a density of 1 × 10^6^ cells per well in a 6-well plate, followed by treatment with 200 ng/mL phorbol myristate acetate (PMA, Beyotime, Shanghai, China) for 24 h, inducing differentiation into unpolarized macrophages. Subsequently, 20 nM recombinant human IL-4 (PeproTech, Rocky Hill, NJ, USA) and 20 nM recombinant human IL-13 (PeproTech, Rocky Hill, NJ, USA) were added, and the cells were further cultured for 48 h to obtain M2-TAMs cells. For the co-culture system, we followed the experimental procedures outlined in the studies by Huang et al. and Jiang et al. [48,49]. Briefly, THP-1 cells were seeded in a 6-well plate, while A549 cells were seeded in a transwell chamber (BD Bioscience, San Jose, CA, USA). PMA, IL-4, and IL-13 were added to induce the polarization of THP-1 into M2-TAMs, with or without the intervention of Chrysin. After 48 h, TAMs from the 6-well plate were collected for flow cytometry analysis.

### 4.3. Western Blot

The cells were lysed on ice using RIPA buffer (Beyotime, Shanghai, China) mixed with a proteinase inhibitor cocktail (Thermo Fisher, Waltham, MA, USA). The quantified cell lysates were subjected to SDS-PAGE using a fast protein gel system (Dakewe Biotech, Shenzhen, China), followed by transfer of the proteins onto PVDF membranes (Pall, Ann Arbor, MI, USA). After blocking with skim milk at room temperature for 2 h, the membranes were incubated overnight at 4 °C with primary antibodies. After washing three times with TBST (Boster, Wuhan, China), the appropriate secondary antibodies were added and incubated at room temperature for 2 h. The protein bands were detected and visualized using an enhanced chemiluminescence (ECL) substrate (Tanon, Shanghai, China) and imaged with a Tanon 5200 Multi gel documentation system (Tanon, Shanghai, China). The band intensities were analyzed using ImageJ software (Image J 1.47v, Bethesda, MD, USA) to quantify grayscale values and perform statistical analysis. The following antibodies were used in the experiment: Rabbit anti-iNOS (Proteintech, Wuhan, China), rabbit anti-ARG-1 (Proteintech, Wuhan, China), mouse anti-β-actin (CST, Danvers, MA, USA), rabbit anti-LC3 (Proteintech, Wuhan, China), rabbit anti-p63 (Proteintech, Wuhan, China), rabbit anti-p-mTOR(Ser2448) (Proteintech, Wuhan, China), rabbit anti-mTOR (Proteintech, Wuhan, China), rabbit anti-p-ULK1 (S555) (ABclonal, Boston, MA, USA), rabbit anti-ULK1 (Proteintech, Wuhan, China), rabbit anti-CDK1 (Beyotime, Shanghai, China), rabbit anti-CDK6 (Beyotime, Shanghai, China), rabbit anti-CCNB1 (Beyotime, Shanghai, China), rabbit anti-CCNB2 (Proteintech, Wuhan, China), and mouse anti-Vinculin (Proteintech, Wuhan, China). The corresponding secondary antibodies included anti-mouse immunoglobulin G (IgG) (Proteintech, Wuhan, China) and anti-rabbit IgG (Proteintech, Wuhan, China).

### 4.4. Flow Cytometry Analysis

To identify macrophage phenotypes, a cell suspension with a concentration of 1–5 × 10^6^ cells/mL was prepared. APC-conjugated anti-F4/80 antibodies (eBioscience, San Diego, CA, USA) and PE-conjugated anti-CD206 antibodies (eBioscience, San Diego, CA, USA) were added for staining. The former was used to identify total macrophages, while the latter served as a marker for M2 macrophages [50]. All antibodies were used at a concentration of 5 μg/mL. After 30 min of incubation on ice, analysis was performed using a BD LSRFortessa flow cytometer (BD Biosciences, San Jose, CA, USA), and data was processed using FlowJo V10 software (Tree Star, Ashland, OR, USA).

### 4.5. Cell Viability Validation

For CCK-8 analysis, cells were seeded at a density of 3 × 10^3^ cells per well in a 96-well plate and incubated overnight, following the instructions provided in the CCK-8 assay kit (GLPBIO, Montclair, CA, USA). On the following day, chrysin was added to the wells and, after a treatment duration of 24, 48, or 72 h, assessments were conducted. For the cell counting assay, cells were seeded at a density of 3 × 10^5^ cells per well in a 6-well plate and incubated overnight. On the next day, corresponding treatments were carried out as per instructions, followed by an additional 48 h incubation before cell counting. For clonogenic assays, cells were seeded at a density of 1 × 10^3^ cells per well in a 6-well plate and incubated overnight. The following day, after cell adhesion, drug treatment was administered for 4 h. After 4 h, the medium was replaced with fresh culture medium, and cells were cultured for two weeks. Once distinct colony formation was observed, fixation was performed using 4% paraformaldehyde (NCM Biotech, Suzhou, China), followed by staining with 0.5% crystal violet (Beyotime, Shanghai, China) overnight, and finally imaging and quantification.

### 4.6. Migration and Invasive Detection

For migration detection, cells were plated in 6-well plates and allowed to reach 100% confluency. As a result, scratches were created on the monolayer with a 200 μL pipette tip, followed by appropriate treatments. Photographs of the scratches were captured at 0 h and 48 h using a light microscope (Motic, Xiamen, China). For invasive analysis, transwell plates (BD Bioscience, San Jose, CA, USA) with 8 μm pores were precoated with diluted matrigel (Corning, Manassas, VA, USA) and placed in 24-well plates. Cells were resuspended in serum-free culture medium and then placed into the transwell chambers at a density of 1 × 10^5^ cells per plate, with complete culture medium containing serum added outside the chambers. After 24 h, cells were fixed with 4% paraformaldehyde, stained overnight with 0.5% crystal violet, and observed and photographed under a light microscope.

### 4.7. LC3-mRFP-GFP Lentiviral Transfection

A549 cells were transfected with the HBLV-mRFP-GFP-LC3-puro lentivirus construct (HanBio, Shanghai, China). Puromycin (InvivoGen, San Diego, CA, USA) was used for screening to establish stable cell lines expressing mRFP-RFP-LC3, achieving an over 80% transfection efficiency. In vitro autophagic activities were evaluated by capturing images using an LMS710 confocal microscope to assess autophagic flux.

### 4.8. Target Identification

The SMILES strings of chrysin were retrieved from the PubChem website (https://pubchem.ncbi.nlm.nih.gov/compound (accessed on 13 January 2024)) and uploaded to Phammapper (http://www.lilabecust.cn/pharmmapper (accessed on 13 January 2024)), SwissTargetPrediction (http://www.swisstargetprediction.ch/ (accessed on 13 January 2024)), and SEA (http://sea.bkslab.org/ (accessed on 13 January 2024)) websites to obtain protein targets for Chrysin. The inclusion criteria for targets obtained from the Phammapper database were set as “Norm Fit higher than average value” (using UniProt website (https://www.uniprot.org/ (accessed on 13 January 2024)) to convert UniProt IDs into corresponding gene target names); for SwissTargetPrediction, targets with a probability greater than 0 were included, and all targets obtained from the SEA database were included. The results from the three databases were then combined, and the union was taken to obtain the final gene targets for Chrysin. Next, gene targets related to “Non-small Cell Lung Cancer,” “autophagy”, and “Tumor Associated Macrophages” were searched from the GeneCards Suite (https://auth.lifemapsc.com/Account (accessed on 13 January 2024)), OMIM (https://omim.org/ (accessed on 13 January 2024)), and NCBI (https://www.ncbi.nlm.nih.gov/ (accessed on 13 January 2024)) databases. The inclusion criteria for targets obtained from GeneCards Suite were set as “Relevance score higher than the average score”, while all targets obtained from the OMIM and NCBI databases were included. The results from the three databases were then combined, and the union was taken to obtain the final gene targets. The intersection of the gene targets for the three diseases and chrysin was calculated. The resulting intersecting genes were uploaded to Metascape (https://metascape.org/gp/index.html (accessed on 13 January 2024)), with the species limited to “Homo sapiens” and a threshold of *p*-value < 0.05, to conduct KEGG and Gene Ontology (GO) enrichment analyses.

### 4.9. Molecular Docking

The three-dimensional crystal structures for target proteins were retrieved from the RCSB database (http://www.rcsb.org/pdb (accessed on 22 January 2024)) and saved as a PDBG file. The three-dimensional structure of chrysin obtained from PubMed was in SDF format. The molecules were processed by AutodockTools.5.6 (http://autodock.scripps.edu/resources/tools (accessed on 22 January 2024)) to remove water and add hydrogen. Molecular docking operations were carried out using Autodock Vina V1.5.6 software to screen for active ingredients with good binding activity to the targets based on the docking score affinity. The scores of each combination were calculated, and the results were visualized using PyMol V4.6.0 software for plotting. 

### 4.10. Mice Experiments

All procedures involving mice were approved by the Animal Protection and Use Committee of Ruige Biotechnology (protocol code 20230615-27). Four-week-old male BALB/c-nu/nu mice were purchased from the Guangdong Medical Experimental Animal Center (Guangzhou, China) for the establishment of the xenograft model. TAMs were coinjected with A549 cancer cells (3 × 10^6^) in a 1:3 ratio subcutaneously into the right dorsal side of the mice. The mice were randomly divided into Ctrl group, TAMs group, and TAMs + chrysin group, with six mice in each group. Chrysin (Meilunbio, Dalian, China) was orally administered to the mice at a dose of 30 mg/kg daily [51]. The mice were observed for growth status every other day, and body weight and tumor size (calculated using the formula: 0.52 × length × width^2^) were measured and recorded. After three weeks of feeding, according to humane euthanasia principles, the mice were euthanized, and tissues were collected. The collected tumor tissues were fixed in 4% paraformaldehyde for 12 h, dehydrated, embedded in paraffin, and then cut into 4 μm sections for immunohistochemistry (IHC) and hematoxylin and eosin (H&E) staining experiments. Following animal welfare principles, the experiment was terminated immediately if the animals lost 20% of their body weight, developed signs of cachexia, or if tumor size exceeded 2000 mm^3^.

### 4.11. H&E Staining and IHC Analysis

For H&E staining, the tissue specimens were first immersed in xylene (Guanghua Chemical, Guangzhou, China) twice to remove the paraffin, followed by dehydration using ethanol solutions of different concentrations (100%, 90%, 80%, and 70%). Then, the specimens were stained with bromophenol blue (Leagene, Beijing, China) and eosin (Leagene, Beijing, China). Subsequently, the specimens were dehydrated using distilled water and various concentrations of ethanol solutions (70%, 80%, 90%, and 100%), before being cleared in xylene. The sections were then mounted using neutral resin (Solarbio, Beijing, China), and images were captured under an Olympus BX46 microscope (Olympus, Melville, NY, USA). For immunohistochemistry analysis, the tissue specimens were deparaffinized and dehydrated following a similar process as the H&E procedure. Then, the specimens were subjected to antigen retrieval by boiling in a 10 mM sodium citrate solution (Solarbio, Beijing, China). Subsequently, the specimens underwent a two-step detection kit process (ZSGB-BIO, Beijing, China) according to the manufacturer’s instructions. In simple terms, the specimens were treated with an endogenous peroxidase blocking reagent (ZSGB-BIO, Beijing, China) at room temperature for 10 min, followed by incubation with primary antibodies (LC3, p62, CDK1) at room temperature for 1 h and subsequent exposure to secondary antibodies at room temperature for 30 min. After that, the specimens were visualized using a DAB chromogenic detection kit (ZSGB-BIO, Beijing, China) and counterstained with eosin. Subsequently, the specimens were dehydrated and cleared, as described earlier. Finally, the sections were mounted using neutral resin (Solarbio, Beijing, China), and images were captured under the Olympus BX46 microscope.

### 4.12. Statistical Analyses

Statistical analysis was conducted using SPSS 27.0 software (IBM, Armonk, NY, USA). Statistical significance was compared through independent samples *t*-tests and one-way analysis of variance (ANOVA). The values were presented as the mean ± standard deviation (SD), with *p* < 0.05 being considered statistically significant.

## 5. Conclusions

In conclusion, this study discovered that chrysin suppresses autophagy activation in lung adenocarcinoma cells mediated by TAMs through the regulation of the CDK1/ULK1 pathway, which reverses the growth-promoting effect of TAMs on lung adenocarcinoma. This offers new insights for the development of novel anti-NSCLC drugs and lays the groundwork for further research into the application of chrysin in cancer treatment. Nevertheless, it is important to acknowledge the limitations of this study, including a restricted range of in vitro cell lines utilized for experiments and a relatively small sample size in the animal models. Therefore, future research is required to further validate the potential value of chrysin in the treatment of lung adenocarcinoma and to explore its possibilities for clinical application.

## Data Availability

The datasets supporting the conclusions of this article are included within the article.

## Supplementary Materials

The following supporting information can be downloaded at: https://www.mdpi.com/article/10.3390/ph17040515/s1, Figure S1: The influence of Chrysin on the polarization of M2-TAMs in a co-culture system; Table S1: Results of the KEGG pathway enrichment analysis; Table S2: Docking score.

## Abbreviations

NSCLC	Non-small cell lung cancer
TAMs	Tumor-associated macrophages
TME	Tumor microenvironment
Chr	Chrysin
3-MA	3-methyladenine
ULK1	UNC-51-like kinase 1
CDK1	Recombinant cyclin dependent kinase 1
WB	Western blot
mTOR	Mammalian target of rapamycin
LC-3	Microtubule-associated proteins light chain 3
CCNB1	Cyclin B1
CCNB2	Cyclin B2
CDK6	Recombinant cyclin dependent kinase 6
