# Supplementary figures and images for: Chrysin Inhibits TAMs-Mediated Autophagy Activation via CDK1/ULK1 Pathway and Reverses TAMs-Mediated Growth-Promoting Effects in Non-Small Cell Lung Cancer
Source: Pharmaceuticals (Basel). 2024 Apr 17;17(4):515. doi: 10.3390/ph17040515 (PMC11055150; doi:10.3390/ph17040515)

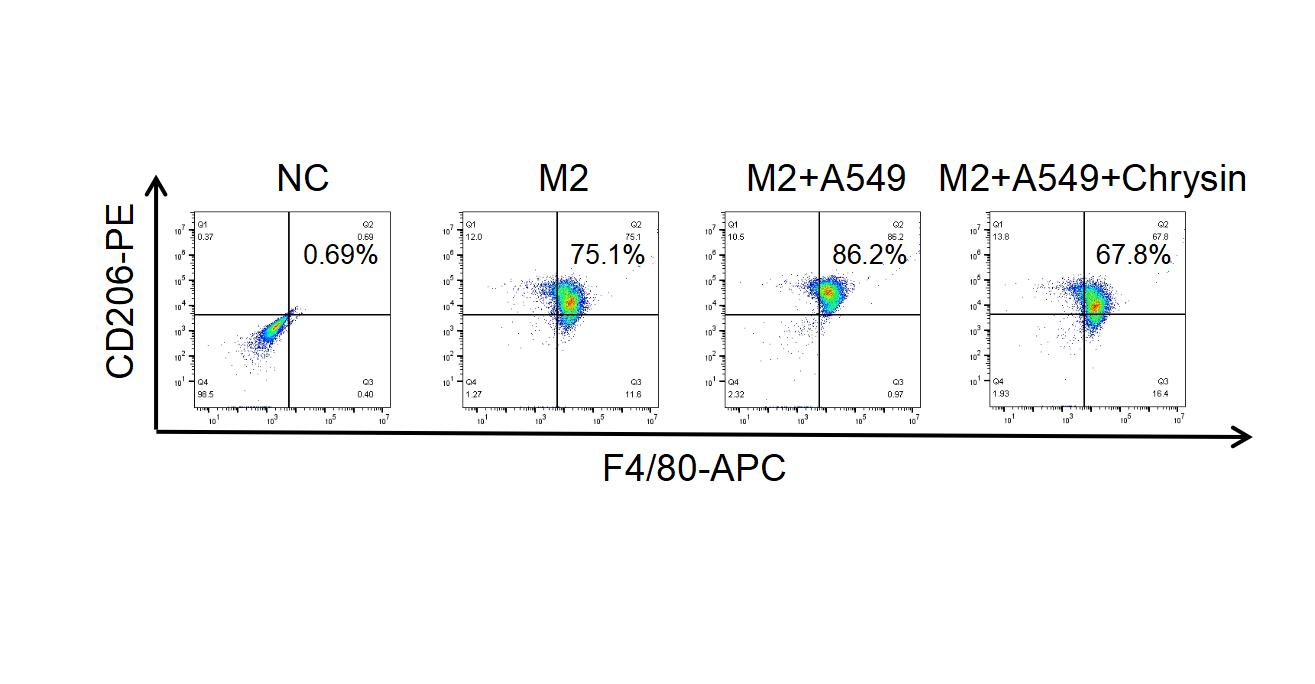

Supplement: Supplementary file 1 [file pharmaceuticals-17-00515-s001.zip › Supplementary figure S1.tif]
